# Supplementary material for: Myeloid TM6SF2 Deficiency Inhibits Atherosclerosis
Source: Cells. 2022 Sep 15;11(18):2877. doi: 10.3390/cells11182877 (PMC9497156; doi:10.3390/cells11182877)
Supplement: Supplementary file 1 [file cells-11-02877-s001.zip › cells-1909076-supplementary.pdf]

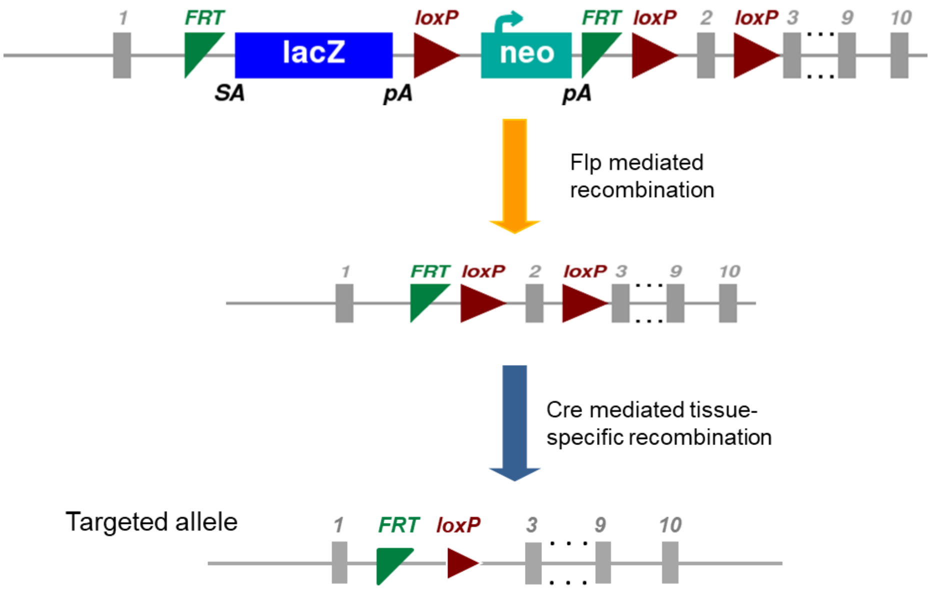

Figure S1. Strategy for conditional disruption of the *Tm6sf2* gene.

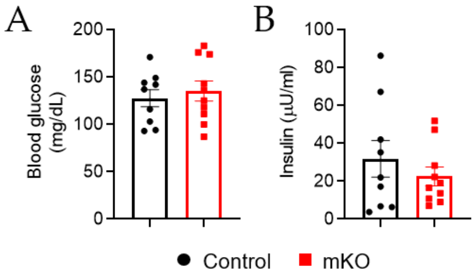

Figure S2. Blood Glucose (A) and Insulin (B) Levels in Myeloid-Specific *Tm6sf2* Knockout Mice.

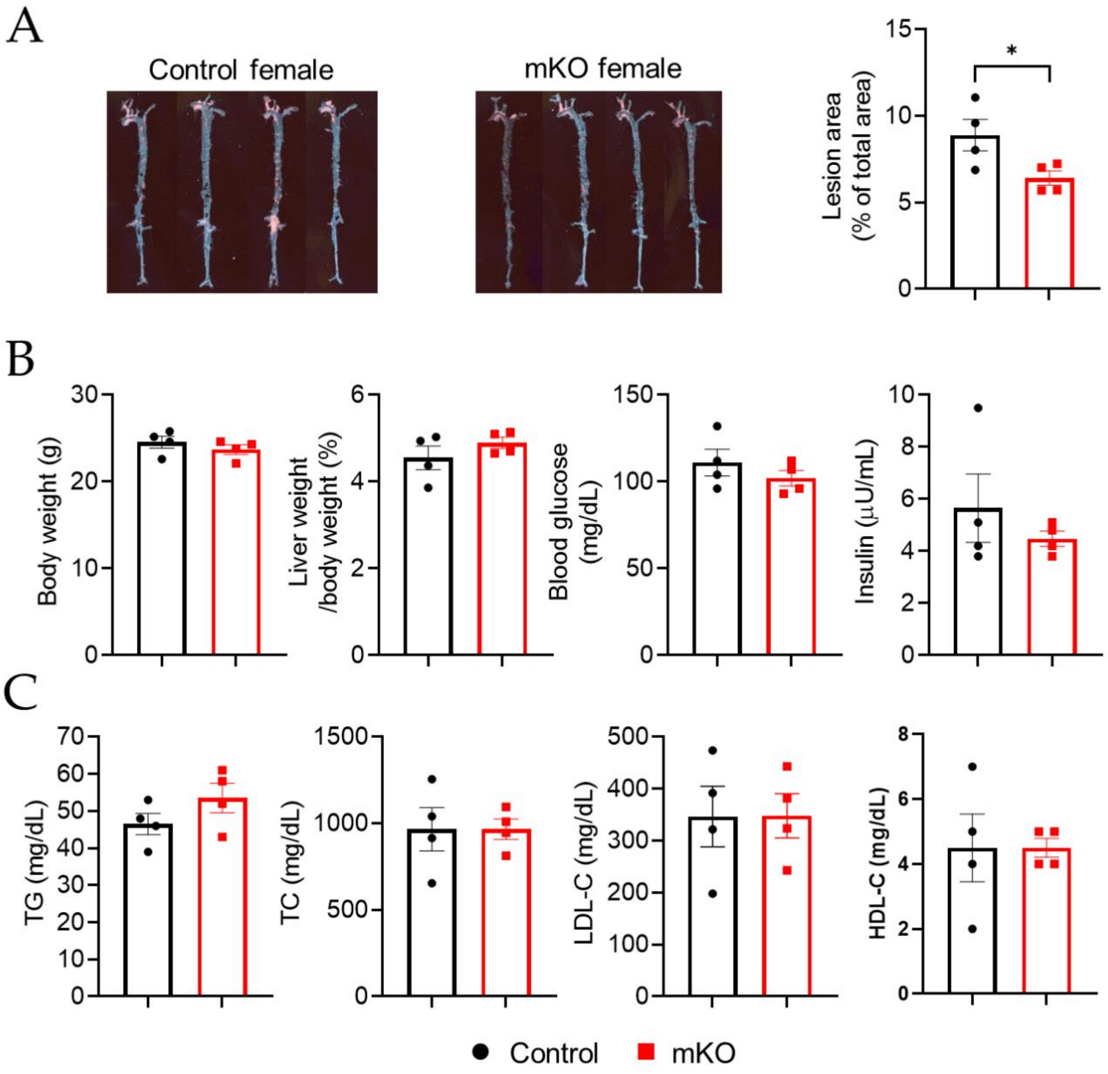

Figure S3. *Tm6sf2* Knockout in Myeloid Cells Inhibits Atherosclerosis Development in Femal Mice. (A) The aortas from control and mKO mice were dissected, stained with Oil Red O, split longitudinally, and pinned open for surface lesion measurements. The lesion area is quantified using Image J. n=4 for each group. (B), Bodyweight, liver weight/body weight ratio, blood Glucose and insulin levels in female control and mKO mice. (C) Circulating TG, TC, LDL-C, and HDL-C levels, n=4 for each group. All data are presented as mean±SEM, * $p<0.05$.

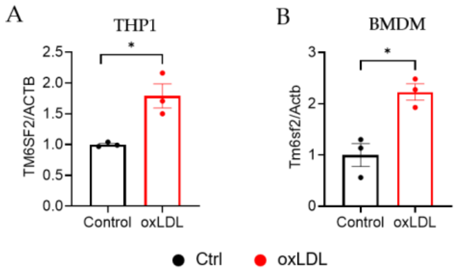

Figure S4. TM6SF2 levels upon oxLDL Treatment. THP-1-derived macrophages (A) or BMDMs were isolated from wild-type C57BL/6J mice were treated with oxLDL (100 μg/ml) for 24 h. mRNA expression of *TM6SF2* was measured by qRT-PCR. n=3 for each group. All data are presented as mean±SEM, * p<0.05.

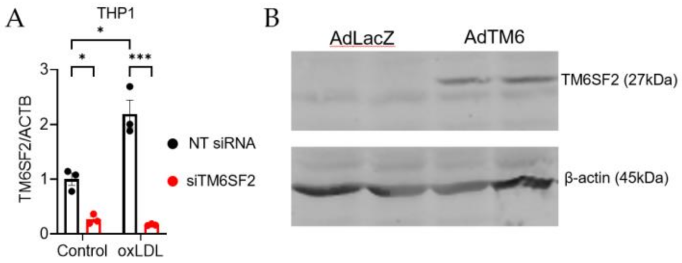

Figure S5. Expression levels of *TM6SF2* in siRNA-induced knockdown or AdTM6SF2-mediated overexpression in macrophages. (A) THP-1-derived macrophages were transfected with NT siRNA or TM6SF2 siRNA (20 nM) for 48 h and treated with oxLDL (100 μg/ml) for 4 h. mRNA expression of *TM6SF2* was measured by qRT-PCR. n=3 for each group. All data are presented as mean±SEM, * p<0.05, *** p<0.001. (B) THP-1-derived macrophages were transfected with AdLacZ or AdTM6SF2 (100 MOI) for 48 h. TM6SF2 level was determined by Western Blotting.

Table S1. Sequences of the primers used for the study.

Gene name	Primer name	seq	application
LysM Cre	oIMR3066	CCC AGA AAT GCC AGA TTA CG	Mutant
	oIMR3067	CTT GGG CTG CCA GAA TTT CTC	Common
	oIMR3068	TTA CAG TCG GCC AGG CTG AC	Wild type
mTm6sf2-Flox	F	AATGTGGGCCTGGGTAGTTTAG	genotyping
	R	GTATTTTCTTGAGGCTCCCAGTTTCT	
Apoe	oIMR0180	GCC TAG CCG AGG GAG AGC CG	Common
	oIMR0181	TGT GAC TTG GGA GCT CTG CAG C	Wild type Reverse
	oIMR0182	GCC GCC CCG ACT GCA TCT	Mutant Reverse
TM6SF2	Tm6sf2 Tg mh-F	TTCTACACCAAGGAGGGAGAGC	TM6SF2 qPCR
	Tm6sf2 Tg mh-R	AACACCAGGATGCTCATGGCGA	
Bip (HSPA5)	h-HSPA5-F1	CATCACGCCGTCCTATGTCG	ER stress gene
	h-HSPA5-R1	CGTCAAAGACCGTGTTCTCG	
	m-HSPA5-F1	ACTTGGGGACCACCTATTCCT	
	m-HSPA5-R1	GTTGCCCTGATCGTTGGCTA	
Ire1a(ERN1)	h-ERN1-F1	CACAGTGACGCTTCCTGAAAC	ER stress gene
	h-ERN1-R1	GCCATCATTAGGATCTGGGAGA	
	m-ERN1-F1	ACACTGCCTGAGACCTTGTTG	
	m-ERN1-R1	GGAGCCCGTCCTCTTGCTA	
ASK1(Map3k5)	h-ASK1-F	CTGCATTTTGGGAAACTCGACT	ER stress gene
	h-ASK1-R	AAGGTGGTAAAACAAGGACGG	
	m-ASK1-F2	CGTGCTGGACCGTTTTTACAA	
	m-ASK1-R2	TGTCACCATGTAGGGGATGAAG	
JNK(Mapk8)	h-JNK-F	TGTGTGGAATCAAGCACCTTC	ER stress gene
	h-JNK-R	AGGCGTCATCATAAAACTCGTTC	
	m-JNK-F	AGCAGAAGCAAACGTGACAAC	
	m-JNK-R	GCTGCACACACTATTCCTTGAG	
XBP1	h-XBP1-F	CCCTCCAGAACATCTCCCCAT	ER stress gene
	h-XBP1-R	ACATGACTGGGTCCAAGTTGT	
	m-XBP1-F	AGCAGCAAGTGGTGGATTTG	

	m-XBP1-R	GAGTTTTCTCCCGTAAAAGCTGA	
TNFa	h-TNFa-F	CCTCTCTCTAATCAGCCCTCTG	inflammation
	h-TNFa-R	GAGGACCTGGGAGTAGATGAG	
	m-TNFa-F	GACGTGGAACTGGCAGAAGAG	
	m-TNFa-R	TTGGTGGTTTGTGAGTGTGAG	
MCP-1 (CCL-2)	h-MCP-1-F	CAGCCAGATGCAATCAATGCC	inflammation
	h-MCP-1-R	TGGAATCCTGAACCCACTTCT	
	m-MCP-1-F	TTAAAAACCTGGATCGGAACCAA	
	m-MCP-1-R	GCATTAGCTTCAGATTTACGGGT	
IL1-b	h-IL1b-F	ATGATGGCTTATTACAGTGGCAA	inflammation
	h-IL1b-R	GTCGGAGATTCGTAGCTGGA	
	m-IL1B-F	GAAATGCCACCTTTTGACAGTG	
	m-IL1B-R	TGGATGCTCTCATCAGGACAG	

m, mouse. h, human
